# In vivo confocal microscopy: qualitative investigation of the conjunctival and corneal surface in open angle glaucomatous patients undergoing the XEN-Gel implant, trabeculectomy or medical therapy

**DOI:** 10.1186/s40662-020-00181-8

**Published:** 2020-03-10

**Authors:** Stefano Baiocchi, Cosimo Mazzotta, Arianna Sgheri, Alessandro Di Maggio, Simone Alex Bagaglia, Matteo Posarelli, Leonardo Ciompi, Alessandro Meduri, Gian Marco Tosi

**Affiliations:** 1 Department of Medicine, Surgery and Neurosciences, Ophthalmology Unit, Policlinico Santa Maria alle Scotte, University of Siena, Viale Bracci 8, ZIP code 53100 Siena, Italy; 2 Ophthalmology Unit, Department of Biomedical Sciences, University of Messina, Messina, Italy

**Keywords:** Glaucoma, Primary open angle glaucoma, POAG, MIGS, Xen 45 gel stent, Ocular surface, Inflammation, Confocal microscopy, IVCM

## Abstract

**Purpose:**

Assessing the quality of the ocular surface by in vivo scanning laser confocal microscopy (IVCM) in primary open angle glaucoma (POAG) patients treated by Xen 45 Gel Stent, medical therapy and trabeculectomy.

**Methods:**

Retrospective, single-center, single-masked, comparative study including 60 eyes of 30 patients (mean age 61.16 ± 10 years) affected by POAG. Eyes were divided into 3 groups: Group 1 eyes underwent the Xen 45 Gel Stent procedure, Group 2 eyes were under medical therapy, Group 3 eyes were surgically treated by trabeculectomy. All patients underwent HRT II IVCM analysis of cornea, limbus, conjunctiva, sub-tenionian space and sclera.

**Results:**

The Xen 45 Gel stent, if properly positioned in the sub-conjunctival space preserves goblet cells and limits ocular surface inflammation. Regular corneal epithelial cells with micro-cysts, and normo-reflective sub-epithelial nerve plexus are documented by IVCM. In sub Tenon’s implants an alternative lamellar intra-scleral filtration is detectable. Combined surgical procedures show a noticeable number of inflammatory cells with rare micro-cysts. Post-trabeculectomy inflammatory reaction is more evident than Xen 45 Gel Stent associated surgical procedures, but less than medical therapy where a conspicuous presence of Langerhans cells, peri-neural infiltrates, marked loss of goblet cells and fibrosis is visible.

**Conclusion:**

Ocular surface inflammation was more notable in topical therapy than after trabeculectomy, which itself causes more inflammation than XEN Gel stents.

## Introduction

Glaucoma represents a group of irreversible, progressive optic neuropathies often leading to severe visual field loss and blindness [1]. Even though intraocular pressure (IOP) elevation is not pathognomonic of glaucoma, its therapeutic management has been considered the standard of care in glaucomatous patients as it is the most modifiable parameter [1].

Different options have been proposed to reduce IOP value such as medical treatment (mono or combination therapy), selective laser trabeculoplasty, filtration surgery with or without antimetabolites or the insertion of aqueous long-tube drainage implants and cyclo-destructive procedures [2].

As the most important and recent guidelines show, medical therapy is one of the first-line approaches in the treatment of primary open angle glaucoma (POAG), exfoliation glaucoma and pigment dispersion glaucoma [2]. It is well known that long-term medical treatments, especially multiple drug associations, induce chronic inflammatory phenomena of the ocular surface with consequent alterations of the Meibomian glands, conjunctiva, cornea and eyelids [3–7].

Generally, when medical treatment does not succeed in stabilizing the progression of the disease and visual field loss, or it is not tolerated for hypersensitive reactions, surgery becomes the only alternative [8]. Trabeculectomy remains the gold standard and the most commonly practiced one [9].

However, despite its efficacy in lowering IOP, trabeculectomy is an invasive surgical procedure with an outcome that is largely influenced by chronic inflammation of the ocular surface due to long-term medical therapy [10]. The most encountered alterations in the conjunctival and corneal epithelium are squamous metaplasia, inflammatory cell infiltrates (granulocytes, lymphocytes, Langerhans cells), dendritic cell activation and loss of goblet cells, and stromal fibrosis [10].

Other common modifications involve nerve plexus alterations and collagen deposition inducing fibrosis [11]. All these chronic inflammatory alterations may negatively influence the surgical outcome thus increasing the possibility of surgical failure by altering the aqueous humor (AH) flow through the bleb wall [12].

Recently, the use of the so-called minimally-invasive glaucoma surgeries (MIGS) is increasing among glaucoma surgeons in order to reduce the invasiveness of conventional filtering surgery and ocular surface toxicity related to long-term anti-glaucoma medical therapies [13, 14]. According to literature, MIGS could be considered a good choice for patients with mild to moderate glaucoma, or for those who are intolerant to standard medical therapy, accounting benefits of minimal trauma with none or only small scleral dissection, minimal or no conjunctival manipulation, an *ab-interno* approach, a good safety profile and faster recovery [15].

In the panorama of MIGS, the use of the Xen 45 Gel Stent (Allergan, Dublin, Ireland) is widely increasing due to its low invasiveness and feasibility [16]. Actually, if well time-planned, MIGS also provide the advantage of operating on a healthy ocular surface and thus increasing the success of filtering surgery [17–20].

The aim of the following study is to compare in vivo laser scanning confocal microscopy (IVCM) qualitative investigation of the ocular surface, including conjunctiva, cornea, limbus, blebs and sclera, in POAG patients who underwent the Xen 45 Gel Stent implant, trabeculectomy or medical therapy.

## Methods

### Study design and patients

This is a retrospective, single-center, single masked, comparative analysis including 60 eyes of 30 adult patients (mean age 61.16 ± 10 years old) affected by POAG, enrolled in the Ophthalmology Department of the Siena University Hospital, Italy (Table 1). All the patients underwent different therapies in each eye: 10 had a Xen 45 Gel Stent in one eye and trabeculectomy in the other, 10 had a Xen 45 Gel Stent in one eye and medical therapy in the other, and 10 had a trabeculectomy in one eye and medical therapy in the other. Eyes were divided into 3 groups: Group 1 included 20 eyes of 20 patients who underwent Xen 45 Gel Stent implantation in one eye. Surgery was performed after several years of topical therapy (38.5 ± 11.9 months) in all patients. Eight patients, already pseudophakic, underwent Xen 45 Gel Stent implantation alone, while 12 patients underwent combined phaco-Xen surgery. All the patients were operated by the same surgeon (SB). All the surgeries were performed with the use of Mitomycin (MMC) 0.2 mg/mL injected in the sub-conjunctival space. No revisions were needed at the time of enrolment. Group 2 included 20 eyes of 20 patients treated with eye-drop medical therapy in one eye for at least 2 years (29.4 ± 7.3 months). Patients were treated by monotherapy with preservative-free Latanoprost 0.005%, or associations of Latanoprost 0.005% plus Timolol 0.5%. Group 3 included 20 eyes of 20 patients who underwent phaco-trabeculectomy in one eye. All the patients underwent trabeculectomy after several years of topical therapy (31.6 ± 9.7 months). All the patients were operated by the same surgeon (SB) using MMC 0.2 mg/mL. All the patients underwent phaco-trabeculectomy and used at least 6 months of post-operative local steroids.

Most patients underwent surgery because medical therapy was unable to guarantee target pressure maintenance, with a consequent visual field impairment progression. Five patients (8%) underwent surgery due to intolerance of topical therapy, without the necessity of reintroducing a topical medical therapy.

### Inclusion criteria

Inclusion criteria were POAG patients with medicated post-operative IOP ≤ 21 mmHg in all cases monotherapy with preservative-free Latanoprost 0.005% or Latanoprost 0.005% plus Timolol 0.5% association (Group 2).

### Exclusion criteria

Exclusion criteria included introduction of antiglaucomatous medication after surgery for groups 1 and 3, pre-existing or concomitant corneal pathologies, post-op complications (ocular hypotony or bleb leakage), history of previous ocular surgery with the exception of cataract phacoemulsification, infectious keratitis, neovascular glaucoma, pigmentary glaucoma, allergic mucosal pathologies and any patient who did not meet inclusion criteria requirements.

The study was approved by the Institutional Review Board of the University of Siena following the tenets of the declaration of Helsinki. All patients signed a specific informed consent form at the time of inclusion in the study and underwent a complete eye examination, including corrected distance visual acuity, IOP measurement with a Goldmann applanation tonometer (Haag-Streit, Koniz, Switzerland), gonioscopy, ocular fundus examinations and IVCM of the ocular surface (cornea, conjunctiva, limbus, sclera, bleb) by means of a HRT II Confocal Microscope (Rostock Cornea Module, Heidelberg Engineering GmbH, Heidelberg, Germany). The clinical and confocal evaluations were performed at a 1-year follow-up from each surgical procedure and at least 1-year after introduction of medical therapy.

### In vivo laser scanning confocal microscopy

All IVCM examinations were performed by the same operator (AD) and confocal scans evaluated by a second blinded independent expert cornea specialist (CM) who looked at the corneal and conjunctival epithelium, corneo-conjunctival junction, corneal sub-epithelial nerve plexus, sub-conjunctival plane, endothelium and bleb’s filtration. In order to improve the reproducibility and the standardization of the images, all corneal scans were performed at the corneal apex, while the conjunctival ones were performed on the bleb (superior sector for trabeculectomy and superior-nasal sector for Xen 45 Gel Stent) and at corneo-conjunctival epithelial junction. Examinations were performed under topical anesthesia with 0.4% oxybuprocaine chlorohydrate (Benoxinate, Alpha Intes, Casoria, Naples, Italy) instilled in the lower conjunctival fornix 2 min before examination. Proper alignment and positioning of the head were maintained with the help of a mobile fixation light for the contralateral eye. A digital camera mounted on the side-arm provided a lateral view of the eye, thus checking in real time for correct alignment. A gel drop of dexpanthenol 5% (Recugel, Bausch & Lomb, Rochester, New York, USA) served as coupling medium between the polymethylmethacrylate contact cup of the objective and the ocular surface.

### Image selection and analysis

Image selection was done by one expert IVCM operator following a format of layer by layer observation. The operator selected the most representative and focused images and rejected the ones with artifacts.

Corneal inflammation was evaluated by the presence/absence of hyper-reflective cells in corneal superficial and basal epithelium, reflectivity rate of cellular network in the basal layer, presence/absence of nuclear activation, number of ramification of sub-epithelial nerve plexus (by choosing in the image with the highest one), tortuosity of the nerve plexus, and the shape of the endothelial layer (presence of polymorphism or polymegathism, and the presence/absence of hyper-reflective spots). Conjunctival analysis included epithelial cell mosaic, goblet cell presence/absence, fibrosis, presence/absence and shape of conjunctival microcysts and bubbles.

### Xen 45 gel stent

The Xen 45 Gel stent is a soft permanent device, shunting AH from the anterior chamber to the sub-conjunctival space. It consists of a 6 mm long flexible tube with a 45 μm diameter lumen made of porcine cross-linked collagen. The device is based on the principles set in the Hagen-Poiseuille equation, establishing that a longer thinner tube will provide more resistance to flow than a shorter and wider one [21].

It is implanted *ab-interno* through a small, self-sealing corneal incision using a preloaded, disposable injector. The surgical procedure is generally performed under indirect observation using a 45° gonioscopic lens according to the surgeon’s experience. The surgeon marks the intended area of placement in the supero-nasal quadrant, which is 3 mm from the limbus [22, 23]. The Xen 45 Gel stent should be implanted anterior to Schlemm’s canal in order to avoid blood reflux into the AC that could occur by needle passage through the above mentioned canal. The procedure may be performed with or without adjuvant antimetabolites (MMC), which in this case would be injected beneath the conjunctiva [24, 25].

## Results

**Fig. 1:**
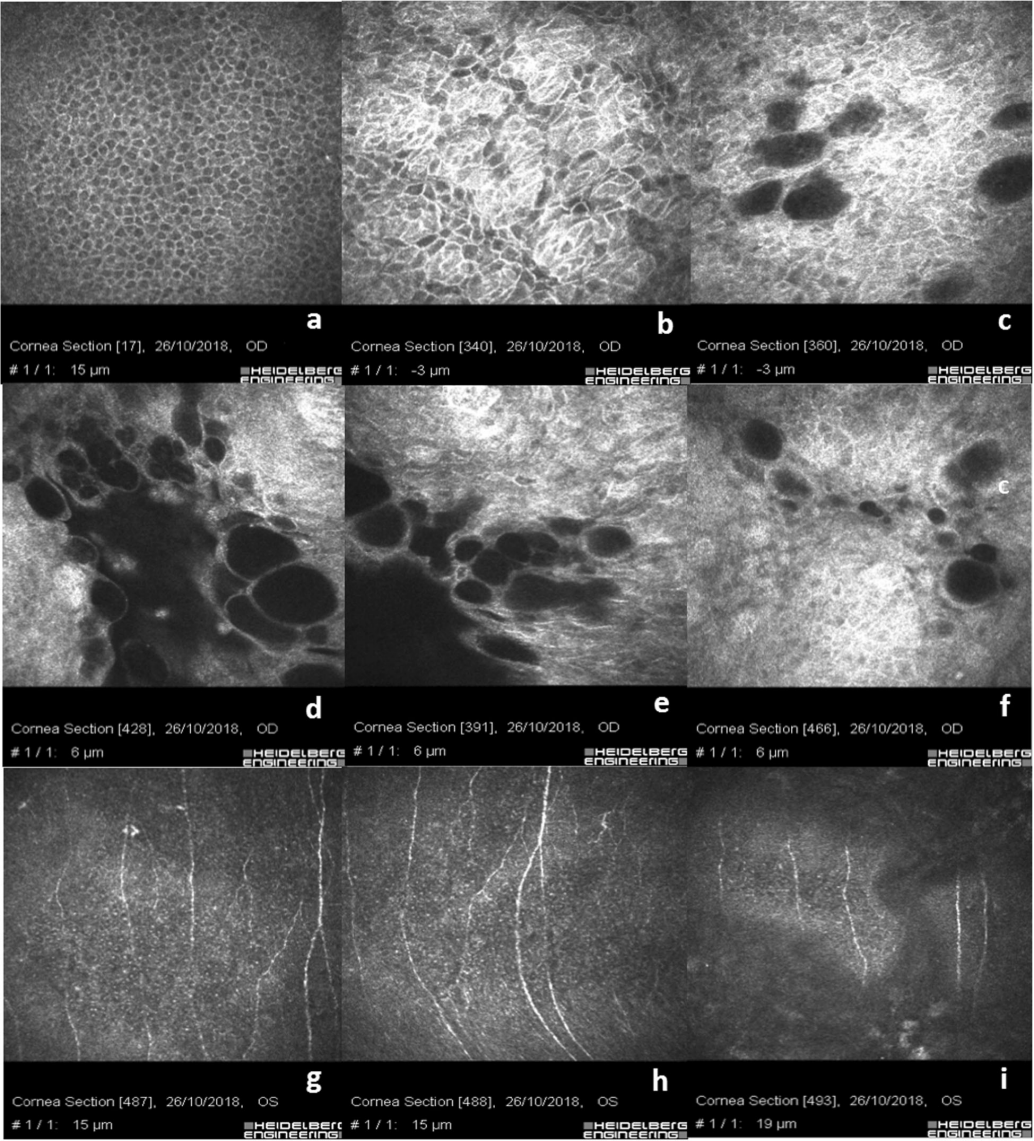
Xen 45 Gel Stent positioned in the sub-conjunctival space. **a** shows regular epithelial basal cells mosaic without rarefaction. **b** shows the junction between conjunctival and corneal basal epithelium in absence of inflammatory cells. **c**-**f** show micro-cysts, microbubbles and bubble clusters aggregate with regular borders in the absence of inflammatory cells. Goblet cells are present in the conjunctival epithelium around the micro-cystic space. **g** shows the sub-epithelial nerve plexus at limbal junction with a slight rarefaction of the nerve fibers and few bead-like neural processes. **h** & **i** show a regular normo-reflective sub-epithelial nerve plexus in the central cornea

The Xen 45 Gel Stent positioned in the sub-conjunctival space as shown in Fig. 1a, shows a regular corneal epithelial basal cell mosaic without rarefaction. The junction between the conjunctival and the corneal basal epithelium is well-documented (Fig. 1b) showing clustered islands of conjunctival epithelium with hyper-reflective cell borders and cytoplasm compared to corneal basal epithelial cells, in the absence of inflammatory cells. Micro-cysts, microbubbles and bubble clusters aggregates with regular borders in the absence of inflammatory cells are well documented (Fig. 1c-f). The conjunctival epithelium appears hyper-reflective at the corneo-conjunctival junction surrounding the micro-cystic blebs (Fig. 1c). Scan g shows the sub-epithelial nerve plexus at limbus junction with a slight rarefaction of the nerve fibers and few bead-like neural processes. Scan h and i show a regular normo-reflective sub-epithelial nerve plexus in the central cornea.

**Table 1:** Study Groups description

	Group 1	Group 2	Group 3
Number of eyes	20	20	20
Number of Patients	20	20	20
Sex			
Male	10	10	10
Female	10	10	10
Pre-enrolment topical therapy duration (months)	38.5 ± 11.9	29.4 ± 7.3	31.6 ± 9.7
Combined Surgery	12	/	20

Group 1: Xen 45 Gel Stent; Group 2: Medical therapy; Group 3: Trabeculectomy

**Fig. 2:**
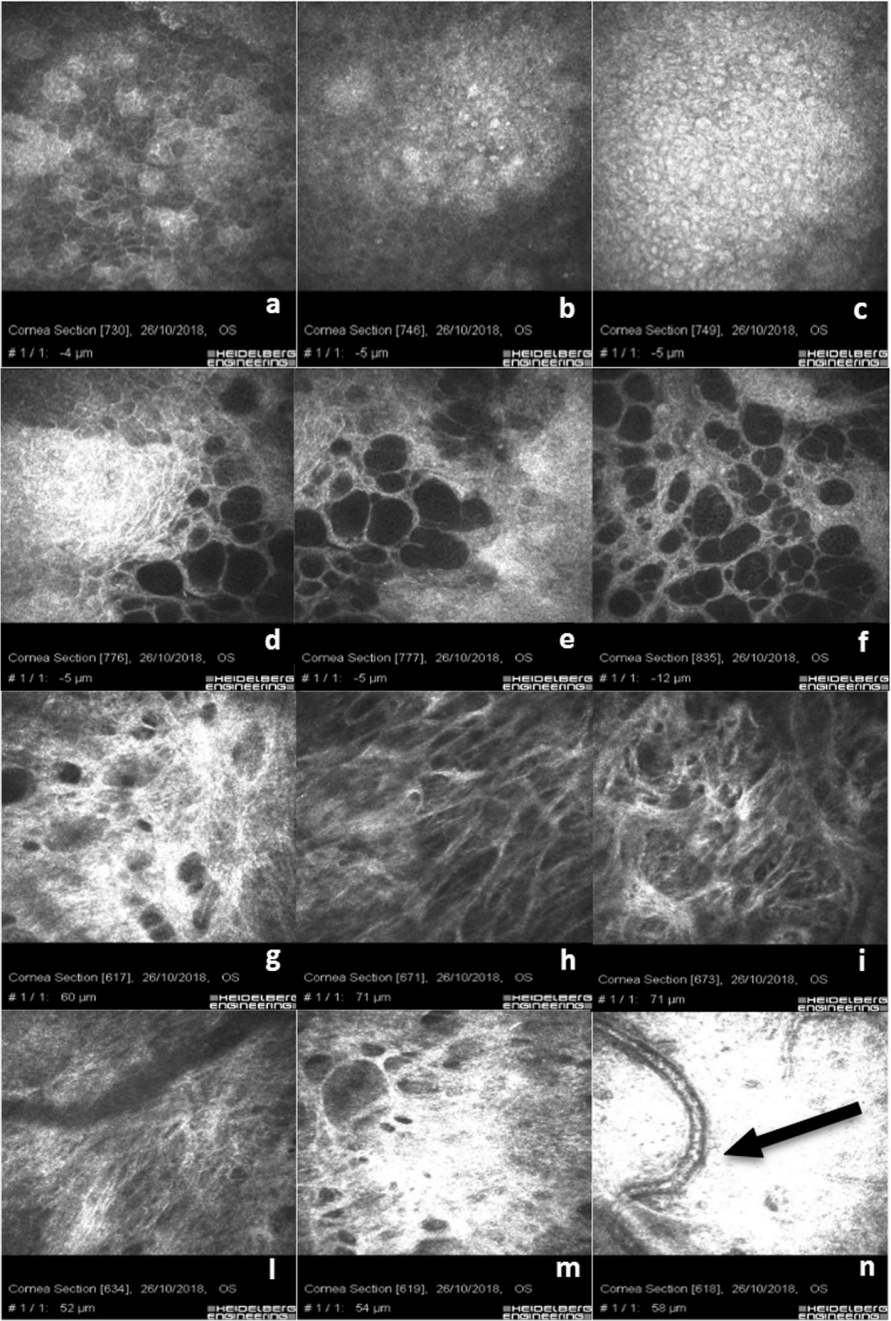
Xen 45 Gel Stent sub-conjunctival filtration. **a** shows the junction between conjunctival and corneal basal epithelium. **b** & **c**) reveal a conjunctival epithelium reach of hyper-reflective goblet cells. **d** shows the corneo-conjunctival junction with blebs and micro-cysts in absence of inflammatory response. **e** & **f** show a regular sub-conjunctival gel stent filtration. **g**, **h**, **i** & **l** show that the sub-conjunctival filtration approaching the tenonian and the scleral planes becomes more laminar than micro-cystic. **m** & **n** show the scleral hyper-reflective plane with basal mixed filtration (micro-cystic and laminar) also documenting a regular vascular loop (black arrow)

Figure 2a shows the junction between corneal and conjunctival hyper-reflective epithelial cells at the boundary of the filtration bleb without inflammatory cells. Scans b, c, d, e and f show typical micro-cysts, hypo-reflective microbubbles and bubble clusters aggregate with regular borders in the absence of inflammatory cells. The presence of goblet cells is relevant and well-visible in the conjunctival epithelium around the micro-cystic space in Fig. 2c and d. Scan g shows the sub-epithelial nerve plexus at limbus junction with a slight rarefaction of the nerve fibers and few bead-like neural processes near the emergency of the needle guide to introduce the stent**.** Scan h and i show a regular normo-reflective sub-epithelial nerve plexus in the central cornea. Figure 2d-f show the corneo-conjunctival junction with hypo-reflective blebs and micro-cysts in the absence of an inflammatory response and hyper-reflectivity of the basal conjunctiva, also documenting an appropriate and regular sub-conjunctival gel stent filtration. Scan g documents the hyper-reflective conjunctival basal plane above the internal space of the filtration bleb. Figure 2h-l show that the sub-conjunctival filtration, approaching the sub-Tenon’s and the deeper pre-scleral and scleral plane, becomes more laminar than micro-cystic. Scans m and n show the scleral hyper-reflective plane with basal mixed filtration (micro-cystic and laminar), also documenting the presence of a vascular loop, as indicated by the black arrow in Fig. 2n.

**Fig. 3:**
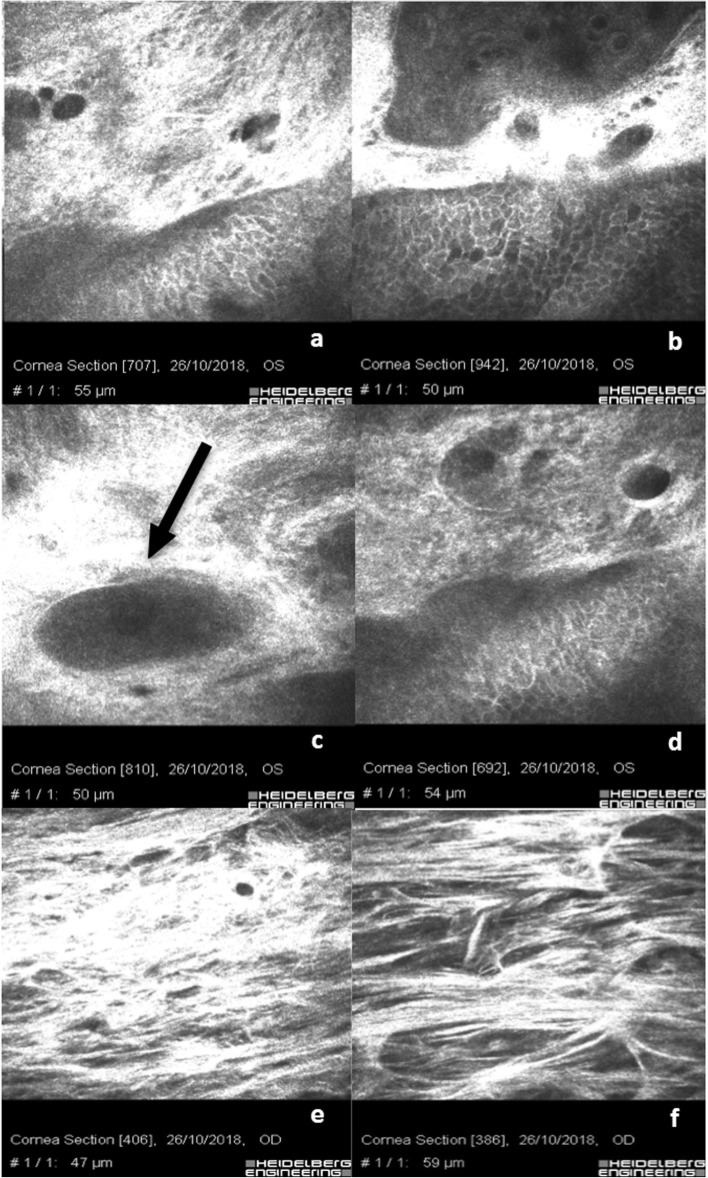
Xen 45 Gel Stent positioned in the sub-Tenon’s space: **a** & **b** show rare filtrating micro-cysts at corneo-conjunctival junction surrounded by a dense hyper-reflective tissue. Goblet cells are more rarefactioned, reflective and not well distinguishable; **c** shows the cross-sectional image of the stent (black arrow); **d** shows irregular epithelial mosaic with rarefaction of basal cells and poor filtration. **e** & **f** clearly indicate a laminar sub-tenonian and intra-scleral filtration and the forced aqueous humor separating the connective lamellae

In 3 patients, the Xen 45 Gel Stent was postoperatively found under Tenon’s capsule. IVCM scans in this case, Fig. 3a & b, show rare filtrating micro-cysts at the corneo-conjunctival junction surrounded by dense hyper-reflective tissue. Goblet cells are rare and not well-distinguishable among the hyper-reflective sub-conjunctival tissue; Fig. 3c shows the cross-sectional image of a Xen 45 Gel stent (Fig. 3c, black arrow); Fig. 3d shows an irregular epithelial hyper-reflective conjunctival mosaic with rarefaction of basal cells and an alternative, even restricted, sub-Tenon’s filtration. Scans e and f clearly show laminar sub-tenonian and intra-scleral filtration and the forced aqueous humor separating the connective lamellae. In these eyes, we evidenced an increase of space separating the scleral meshwork.

**Fig. 4:**
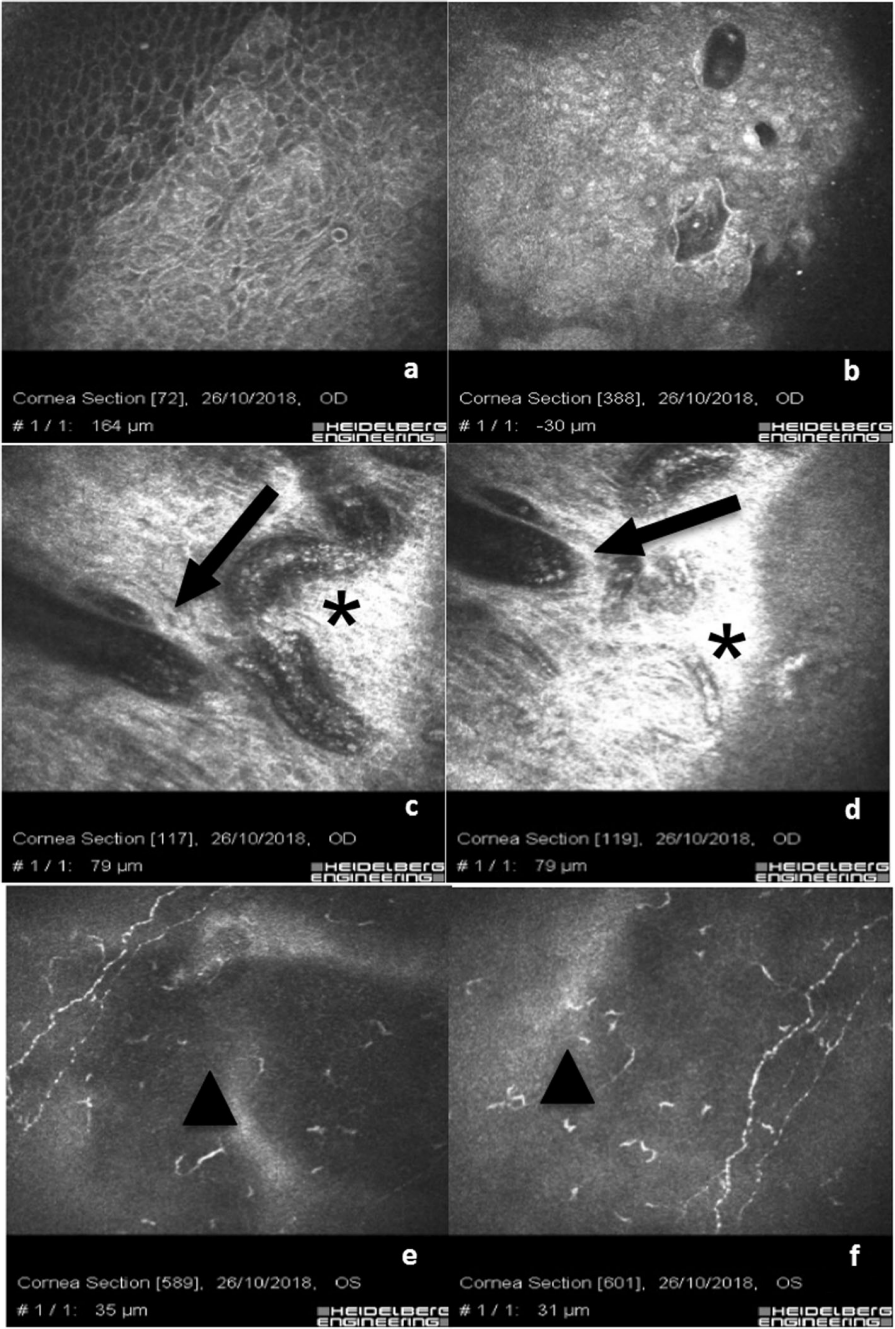
Combined Xen 45 Gel Stent plus Phacoemulsification: **a** & **b** show the corneo-conjunctival junction with rare blebs, preserved goblet cells and intra-cystic bright micro-particles maybe of pigment or inflammatory origin. **c** & **d** show the Xen 45 Gel stent (black arrows) positioned in the sub-tenonian space containing bright micro-particles of potential pigment origin, surrounded by hyper-reflective tissue and blood vessels (black asterisks). **e** & **f** show the presence of Langerhans cells (black triangles). A peri-neural corneal inflammation was observed in all patients where the Xen 45 Gel Stent was combined with cataract surgery compared with Xen 45 Gel Stent alone. The sub-epithelial nerve fibers appeared hyper-reflective assuming a bead-like shape

In the eyes where the implant of the Xen 45 Gel Stent was performed in combination with phacoemulsification, as shown in Fig. 4, we observed a conspicuous number of conjunctival inflammatory cells. Figure 4a & b show the corneo-conjunctival epithelial junction with rare blebs and micro-cysts, preserved hyper-reflective goblet cells and intra-cystic bright micro-particles, perhaps of pigment or inflammatory origin (presumably granulocytes). Figure 4c & d show the gel stent (black arrows) positioned in sub-Tenon’s space containing bright micro-particles of potential pigment origin, surrounded by hyper-reflective tissue and blood vessels (black asterisks). Scans e and f show the presence of Langerhans cells (black triangles). A peri-neural corneal inflammation was observed in all patients where the Xen 45 Gel Stent was combined with cataract surgery compared with the Xen 45 Gel Stent alone. The sub-epithelial nerve fibers appeared hyper-reflective assuming a bead-like shape.

**Fig. 5:**
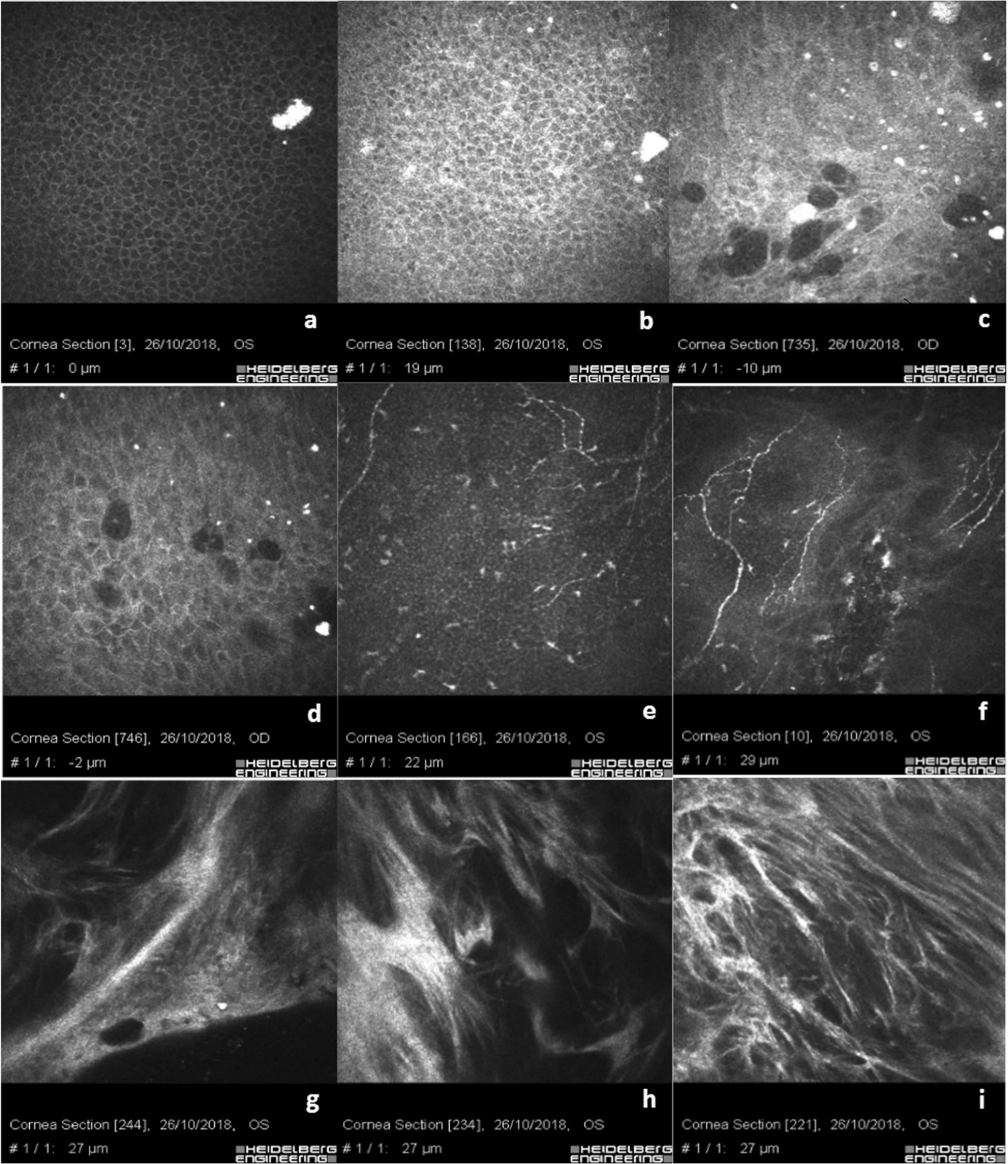
Trabeculectomy: chronic inflammatory corneo-conjunctival infiltration. **a**-**c** show basal epithelium after trabeculectomy with diffuse hyper-reflective inflammatory cells (infiltrate) and bright micro-particles. Micro-blebs are evident associated with lymphocytes and pigment at basal epithelium in (**d**). **e** & **f** show a rarefaction of sub-epithelial nerve fibers associated with peri-neural inflammation. **g**-**i** show the bleb with intra-bleb connective tissue sepiments indicating a potential time-dependent inflammatory fibrosis and bleb failure

Eyes of patients who underwent trabeculectomy were characterized by the presence of a well-documented ocular surface with chronic inflammation. Figure 5a-c show basal corneal epithelium after trabeculectomy with a hyper-reflective cell border and cytoplasm (activation), diffuse hyper-reflective inflammatory cells (presumably of lymphocyte and granulocyte origin) associated with bright micro-particles. Micro-blebs are evident and associated with lymphocytes and pigment at basal epithelium in Scan d. Scans e and f show clear rarefaction of the sub-epithelial nerve fibers associated with peri-neural inflammation (infiltrate). Scans g, h and i show the bleb with intra-bleb connective tissue segments indicating a potential time-dependent inflammatory fibrosis and bleb failure. Post trabeculectomy inflammatory reaction, as documented by IVCM analysis, was much more evident than Xen 45 Gel Stent associated surgical procedures, but less than medical therapy as shown in Fig. 5. There were no morphological differences between bleb microstructure between a sub-conjunctival Xen 45 Gel stent implant and a well filtering trabeculectomy at the epithelial level; while in the deeper sub-conjunctival layers the density of mycrocists and blebs was more evident in the Xen 45 Gel stent while the structure of the trabeculectomy bleb showed larger and less clustered spaces.

**Fig. 6:**
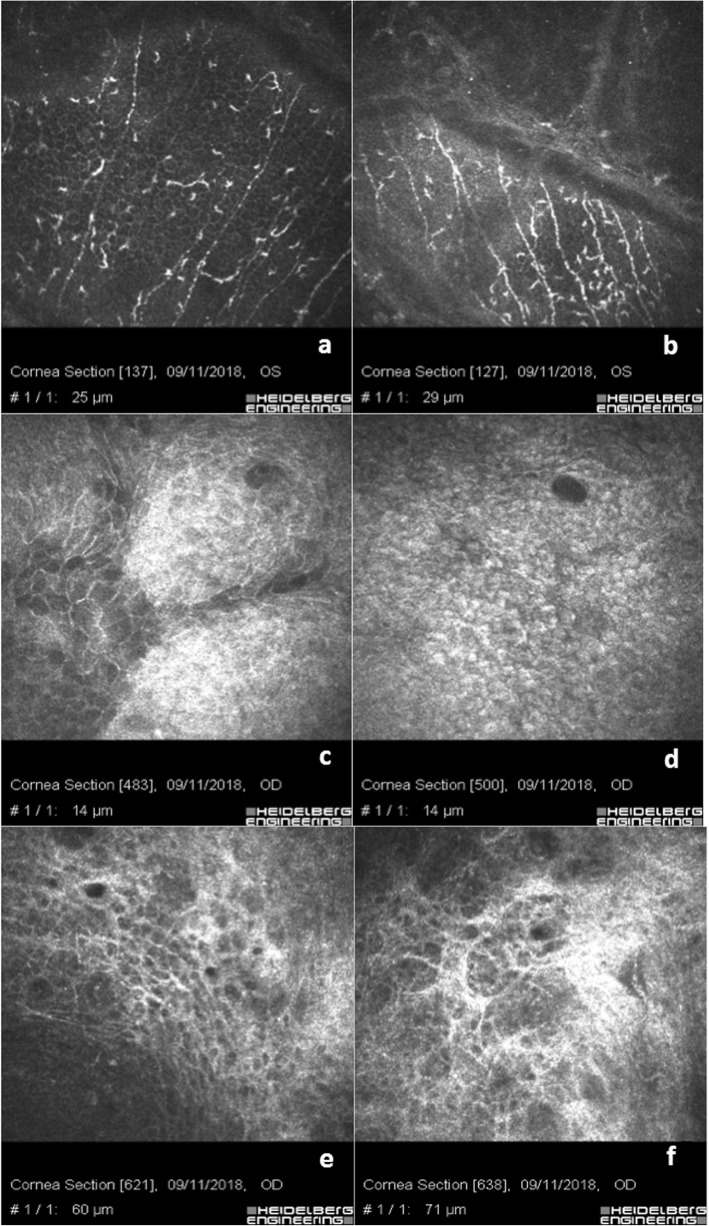
Chronic corneo-conjunctival inflammation after topical medical therapy: **a** & **b** show a severe inflammation of the ocular surface with Langerhans cells, rarefaction of nerves fibers and peri-neural infiltrates. **c** & **d** show the corneo-conjunctival junction with alteration of epithelial cells mosaic, irregular cell bordered and extreme rarefaction of goblet cells. **e** & **f** show the loss of goblet cells with hyper-reflective fibrosis of the ocular surface

In eyes with medical therapy we documented a higher density of ocular surface chronic inflammatory reaction. As shown in Fig. 6a and b, the severe inflammation of the ocular surface was proven by a conspicuous presence of Langerhans cells, rarefaction of nerve fibers and peri-neural infiltrates. Scans b and c show the corneo-conjunctival junction with alteration of epithelial cell mosaic, irregular hyper-reflective cell borders, marked loss of goblet cells and fibrosis. Scans e and f show the loss of goblet cells with hyper-reflective fibrosis of the ocular surface.

**Fig. 7:**
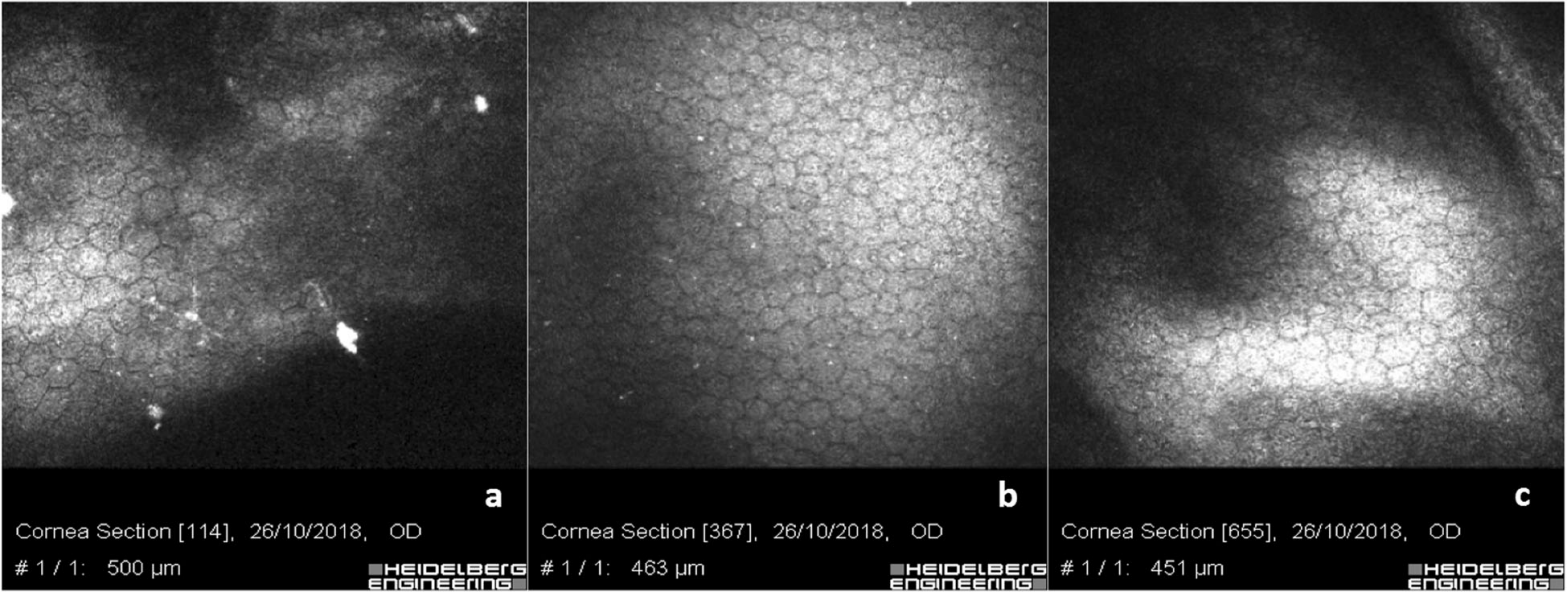
Endothelium after XEN 45 Gel Stent, Trabeculectomy, Medical Therapy: **a** shows corneal endothelium after XEN gel stent implant with a regular mosaic but include bright particles that could be deposits or lymphocytes, indicating a potential retrograde flow through the stent from the sub-conjunctival space to the anterior chamber. **b** shows a regular endothelium after trabeculectomy with tiny bright micro-particles of pigment origin. **c** shows endothelial mosaic with slight polymorphism and polymegathism in patients with medical therapy

As shown in Fig. 7, the analysis of corneal endothelium showed a regular cell mosaic but included some bright particles that could be deposits or lymphocytes indicating a potential retrograde flow through the stent from the sub-conjunctival space to the anterior chamber post Xen 45 Gel Stent implantation (Fig. 7a). A regular endothelial mosaic was documented in trabeculectomy patients with some tiny bright micro-particle deposits, reasonably of pigment origin (Fig. 7b). Scan c shows the endothelial mosaic with slight polymorphism and polymegathism in patients with medical therapy.

## Discussion

The main goal in glaucoma therapy is reaching a personally targeted IOP in order to prevent or stop the loss of visual field in both hypertensive or normotensive glaucomatous patients [26]. The aim of the treatment is to prevent, or avoid, visual field deterioration and reach and maintain the target IOP, taking into account quality of life (QOL) and life expectancy of the patient. According to international guidelines [2], the individualized IOP target can be reached by different means, alone or combined through medical therapy, para-surgical and surgical approaches. Unfortunately, all these options can lead to ocular surface alterations due to chronic inflammation. Topical medical therapy is the main cause of conjunctival and corneal microstructure alterations. It is widely known that both preserved and preservative-free (PF) drugs induce modifications on the ocular surface and adnexa [27–30], thus causing ocular discomfort, low QOL and poor patient compliance. Different studies [31, 32] demonstrated that excipients and preservatives play a primary role as “*drivers*” of chronic inflammation of the ocular surface by inducing hypersensitive reactions or, more frequently, a persistent cytotoxic response, also leading to instability of the pre-corneal tear film. Mastropasqua et al. [17–20] found a wide spectrum of ocular surface modifications such as squamous metaplasia, desquamation, keratinization, dendritic cells activation and goblet cells loss in corneal and conjunctival epithelium, while the scleral stroma was characterized by a hyper-reflective signal, especially in patients undergoing long-term topical therapy. In our experience, as shown in Fig. 6, we evidenced the loss of goblet cells, pleomorphism of epithelial cell mosaic and irregular hyper-reflective cell borders (Scans c and d) with hyper-reflective fibrosis of the sub-conjunctival meshwork (Scans e and f) in Group 2, in line with findings described by Ciancaglini et al. [5] A high level of inflammatory infiltration, mostly constituted of dendritic-like cells (probably Langerhans cells), was present in the corneal sub-epithelial layer and in particular close to nerve ramifications that were less ramified, as visible in Fig. 6a and b. These findings were similar to those of Martone et al. [33] In patients with uncontrolled IOP by topical medical agents and with progressive visual field impairment, trabeculectomy represents the gold standard of surgical therapy. Different studies demonstrated that trabeculectomy lowers IOP efficiently, allowing better control of mean 24-h pressure, but it widely modifies ocular surface anatomy, causing a persistent clinical or subclinical inflammatory process [9, 30]. In line with the literature [34] our experience demonstrates that eyes that underwent trabeculectomy were characterized by the presence of well-documented ocular surface chronic inflammation as showed in Fig. 5, with hyper-reflective cell borders and cytoplasm (activation) of corneal basal epithelial cells, diffuse hyper-reflective inflammatory cells (presumably of lymphocyte and/or granulocytes origin) infiltrating the conjunctival epithelium as documented in Fig. 5a-c). Epithelial micro-cysts can easily be revealed by IVCM and have been described as a mark of trans-conjunctival outflow of the AH [5]. The high number of micro-cysts revealed in our series indicated an optimal filtration, in line with the literature [34, 35].

Late-functioning blebs in our records showed the presence of connective tissue sepiments within the bleb, indicating a reasonable time-dependent inflammatory fibrosis and a potential future bleb failure (Fig. 5g-i). Scans e and f show a clear rarefaction of the sub-epithelial nerve fibers associated with peri-neural inflammation infiltrate.

The recent introduction of the Xen 45 Gel Stent in the MIGS panorama offers one more option in hypertensive glaucoma management in patients with medium to moderate grade glaucoma. Conjunctival sparing in Xen 45 Gel Stent implant combined with a placement technique that respects local anatomy guarantees less stress on the ocular surface, reducing inflammatory triggers [33, 36]. There are few reports in literature regarding IVCM Xen 45 Gel Stent analysis. One of the few studies on this topic was published by Fea et al. [37], describing an increased density of epithelial microcysts with a reduction of density of subepithelial connective tissue. Our IVCM analysis of eyes with Xen 45 Gel Stents positioned in the sub-conjunctival space was characterized by a regular shape of corneal basal epithelium as showed in Fig. 1a and with few hyper-reflective dots in the superficial layers. The corneal sub-epithelial nerve plexus at the central cornea was normo-reflective, with a slightly parallel pathway and a low grade of ramification (Fig. 1g-i). This sign could be related to the lower global rate of ocular surface inflammation that we found in patients who underwent minimally invasive surgery.

Martone et al. [33] described a reduction of the number of fibers and reflectivity of the sub-basal plexus, associated with a higher tortuosity of nerve ramifications in medically treated glaucomatous patients. Healthier conjunctival epithelium status is proved by the presence of goblet cells particularly visible around the micro-cystic spaces (Fig. 2c-e).

Micro-cysts, microbubbles and bubble clusters were largely recognizable and characterized by regular borders, without infiltration of inflammatory cells (Fig. 1c-f; Fig. 2d-f). The sub-conjunctival tissue was loosely structured and characterized by the presence of mixed filtration (micro-cystic and laminar), (Fig. 2h, i, m & n). IVCM analysis helped us identify an optimal or partial surgical outcome showing, in three eyes with the Xen 45 Gel Stent positioned in the sub-tenonian plane, an irregular and hyper-reflective epithelial conjunctival mosaic with basal cells rarefaction and an alternative, even partially impaired, sub-Tenon’s filtration. A small number of micro-cysts combined with altered intra-scleral lamellar filtration were detected in these patients. This aspect is reasonably due to AH forced to follow an alternative outflow pattern, inducing a lamellar intra-scleral filtration pathway (Fig. 3e & f).

In eyes that underwent phacoemulsification cataract surgery combined with the Xen 45 Gel Stent implantation, we observed a significant number of conjunctival inflammatory cells, made of tiny hyper-reflective elements, presumably of lymphocyte origin**.** At the corneo-conjunctival epithelial junction, it was possible to detect reduced dimension blebs and micro-cysts with preserved hyper-reflective goblet cells and intra-cystic bright micro-particles of probably pigment or inflammatory origin, as visible in Fig. 4a & b.

In Fig. 5e & f, the inflammatory reaction at IVCM analysis was much more evident after trabeculectomy than in all XEN 45 Gel stent procedures, including combined implantations, but less than medical therapy as shown in Fig. 6a & b. There were no morphological differences between bleb’s microstructure between a sub-conjunctival XEN implant and a well filtering trabeculectomy at the epithelial level, while in the deeper sub-conjunctival layers the density of mycrocists and blebs was more evident in the XEN gel stent and the structure of trabeculectomy bleb showed larger and less clustered spaces.

Slight endothelial cell polymorphism and polymegathism was present in the majority of patients (Fig. 7). The most relevant aspect of IVCM endothelial analysis in eyes with the Xen 45 Gel Stent implantation was the presence of bright micro-particles and deposits (Fig. 7a & b), not detectable at the slit lamp examination on the endothelial surface. In our opinion, they could represent proof of a back-flow phenomenon, possibly happening when the pressure gradient is inverted due to a mechanical action insisting on the bleb, such as eye-rubbing encouraged by ocular surface inflammation, foreign body sensation or dry eye symptoms.

Our experience confirmed the presence of a clinical or subclinical chronic inflammation involving both cornea and conjunctiva in patients with either medical or surgical therapy. The novelty of IVCM qualitative analysis was the comparative evaluation between eyes that underwent Xen 45 Gel Stent surgery, patients undergoing medical therapy and patients who underwent phaco-trabeculectomy. Ocular surface inflammation was more notable in topical therapy than after trabeculectomy, which itself causes more inflammation than XEN Gel stents.

## Conclusions

The long-term ocular surface changes induced by various treatment choices in POAG patients demonstrate the potential of the Xen 45 Gel Stent, especially when properly positioned under the conjunctiva, in preserving ocular surface integrity compared to medical therapies and thus the potentiality of improved outcomes for any subsequent surgeries. In this context, the Xen 45 Gel Stent could represent a possible temporary solution in patients where a successful trabeculectomy surgical outcome could be impaired by severe ocular surface inflammation due to long-term topical medical therapy. Anyway the ocular surface inflammation was more notable in topical therapy than after trabeculectomy, which itself causes more inflammation than XEN Gel stents.

## Data Availability

Not applicable.
